# Prolonged severe immunodeficiency following thymectomy and radiation: a case report

**DOI:** 10.1186/1752-1947-8-457

**Published:** 2014-12-21

**Authors:** Johanna Lee Wickemeyer, Sudhir Sekhsaria

**Affiliations:** 15 Georgetown University School of Medicine, 3900 Reservoir Rd NW, Washington, DC, 20007 USA; 16 Medstar Union Memorial Hospital, 3333 N Calvert St, Suite 520, Baltimore, MD 21218 USA

**Keywords:** Chronic idiopathic urticaria, Immunodeficiency, Lymphocytopenia, Thymectomy, Thymoma

## Abstract

**Introduction:**

Immunodeficiency can occur both in patients undergoing radiation therapy, as well as in patients who have had thymectomies. However, few studies have examined the immune recovery of a patient following both procedures. We aim to emphasize the need for assessment and consistent monitoring of patients with thymoma prior to and after combined treatment of thymectomy and radiation, both of which are likely to result in an increased risk for immunodeficiency.

**Case presentation:**

We describe the longitudinal progress of a 59-year-old Asian male who underwent thymectomy followed by radiation therapy and subsequently presented with generalized urticaria. Revelation of a low absolute lymphocyte count (615 cells/mcL) on initial evaluation prompted further analysis of his immunoglobulin levels and antigen response to a polysaccharide pneumococcal vaccine (PneumoVax-23). Although his immunoglobulin levels were unremarkable, he failed to respond to 11 of 12 serotypes of the pneumococcal vaccine. As a result, he was placed on Bactrim^®^ (trimethoprim-sulfamethoxazole) prophylaxis to prevent opportunistic infections, and his CD4+ and CD8+ counts were monitored over the course of 8 years. His lymphocyte counts 87 months after thymectomy and 85 months after radiation therapy were as follows: absolute lymphocyte count 956 cells/mcL, absolute CD3+/CD4+ 164/mm^3^ (16%) and absolute CD3+/CD8+ 257/mm^3^ (25%). The patient was able to discontinue Bactrim^®^ (trimethoprim-sulfamethoxazole) prophylaxis after 9 years of treatment.

**Conclusions:**

The lymphocytopenia, low CD4+ count, and failed response to pneumococcal vaccination that presented in our patient are consistent with immunodeficiency. After radiation alone, a recovery of T-lymphocytes is usually observed after approximately 3 weeks. Over the course of 8 years, he has still not made a full recovery according to laboratory markers, which seem to have stabilized at chronically low levels. To prevent serious complications, we suggest that patients who have undergone both thymectomy and radiation therapy be monitored for immunodeficiency. This case report informs the practices of allergists, oncologists, and neurologists in the continuing care of patients with thymoma.

## Introduction

The thymus, an organ composed of lymphoid and epithelial tissue, is located in the anterior superior mediastinum and responsible for the selection and maturation of T-lymphocytes. On the onset of puberty, the thymus begins to involute, shrinking in size throughout adulthood and leading to a profound decline of thymic function by early adulthood. Thymoma, arising from the epithelial cells, is an uncommon tumor of the thymus and is typically treated with surgical excision. Induction and/or adjuvant chemotherapy may be used if the invasion of the tumor infiltrates into surrounding tissue. A commonly used regimen in current practice is cisplatin-based chemotherapy. However, there are no randomized trials that evaluate the effectiveness of chemotherapy for thymic carcinoma or advanced thymoma in adults [1]. Furthermore, the prognosis of patients with totally resected stage III and stage IV thymoma was not improved with adjuvant therapy [2].

Radiation therapy of the mediastinum causes a rapid decrease in circulating B- and T- lymphocytes. While the acute decrease in lymphocytes post-procedure is short lived, most patients show a modest chronic depression in both numbers and function of circulating lymphocytes [3, 4]. CD4+ lymphocyte recovery after dose-intense chemotherapy is constrained in adults both by a limited thymic regenerative capacity, as well as an increased susceptibility to apoptosis within the expanding peripheral CD4+ population [5, 6]. In a study of children and young adults exposed to chemotherapy, the thymopoietic pathway of CD4+ T-lymphocyte regeneration was shown to be important throughout childhood. However, it is unclear whether there is an age after which the thymus loses it regenerative capacity completely [7].

Thymectomy is a common therapeutic option to treat myasthenia gravis (MG), a condition commonly associated with thymoma. In a long-term study of patients who had undergone a thymectomy, laboratory work demonstrated a mild T-cell lymphocytopenia and an expansion of some V-beta families among the circulating CD4+ and CD8+ T cells, as well as organ and non-organ-specific autoantibodies [8]. A more recent study suggested that extrathymic development of T-cells in the tonsils supports the generation of autoreactive T-lymphocytes, and may even contribute to malignant transformation [9]. Extrathymic T-cell development suggests an increase of systemic autoimmune disease secondary to thymectomy in patients with MG [8], possibly explaining the development of systemic lupus erythematosus (SLE) in patients months or years after surgical thymectomy [10]. This discovery assumes an important role in adults who have undergone chemotherapy, accounting for increased autoimmunity in these patients [11, 12].

In this case report, we present a patient who developed a significant, sustained CD4+ lymphocytopenia and chronic idiopathic urticaria after a combination of thymectomy and radiation as treatment for thymoma.

## Case presentation

Over 9 years ago, a 59-year-old Asian man was referred to our office by his primary care physician with a history of extensive daily hives consisting of severe itching for the past 3 to 4 months. While his skin rash was aggravated by stress and cold weather, there was no association with any foods or medications. The only unusual aspect of his past medical history was a microinvasive thymoma involving the capsule, which was diagnosed 2 years prior to his appointment at our office. Before treatment of the thymoma, his white cell and absolute lymphocyte counts were within normal limits; however, his CD4+ and CD8+ counts were not measured prior to surgery. Six weeks following the thymectomy to remove the tumor, he received mediastinal radiation of 62 gray. His rash began approximately 2 weeks after his thymectomy. Two months following the completion of chemotherapy, he sought care. While his immune function had not been specifically evaluated prior to his thymectomy and radiation, his medical history was negative for any known immune disorders, and he indicated no previous symptoms for immune disorders. Furthermore, his family history was negative for autoimmune disorders. The physical examination on his initial visit was only significant for extensive urticarial rash on his trunk and both extremities with no angioedema.

Two months after the radiation was delivered, our initial laboratory work-up was significant for a depressed absolute lymphocyte count of 465 cells/mcL (normal values, nml: 850 to 3900 cells/mcL). The combination of his low absolute lymphocyte count and non-allergic urticaria prompted further laboratory evaluation of a complete blood count differential, CD4+ and CD8+ counts, thyroid-stimulating hormone (TSH), and thyroid autoantibodies. We diagnosed him as having chronic idiopathic urticaria (CIU) and prescribed fexofenadine 180mg once daily before noon and doxepin 50mg every evening at bedtime.

A subsequent follow-up visit a month later revealed a severe CD4+ lymphocytopenia of 75/mm^3^ (10.9%; nml 490 to 1740/mm^3^, 30 to 61%) with CD8+ cell count at 336/mm^3^ (49%; nml 180 to 1170/mm^3^, 12 to 42%); however, his TSH, thyroid peroxidase, and thyroglobulin antibodies were within normal limits. He tested negative for human immunodeficiency virus. Although his immunoglobulin levels were unremarkable, he failed to respond to 11 of 12 serotypes of the polysaccharide pneumococcal vaccine. To prevent opportunistic infections, we prescribed him Bactrim^®^ (trimethoprim-sulfamethoxazole) double strength once daily prophylaxis. Over the next few weeks, his urticaria improved between 40 and 50%, albeit he continued to experience a daily rash and frequent itching.

For 2 years after his first appointment at our office, he was followed every 4 weeks with close monitoring for any opportunistic infections and every 2 to 3 months with laboratory evaluation for CD4+ and CD8+ counts. Fifteen months following his thymectomy, he developed a severe case of shingles on his trunk and left arm, for which he was treated with Famvir^®^ (famciclovir) 500mg twice daily and Zovirax^®^ (acyclovir) cream twice daily. He continued to have severe, persistent CD4+ lymphocytopenia, reaching a maximum of 130 cells/mm^3^ (nml: 490 to 1740/mm^3^, 30 to 61%) over the course of 2 years post-thymectomy (Table 1).

**Table 1:** **Report of our patient’s absolute lymphocyte counts over 7 years post-thymectomy: CD3+/CD4+ T-cell counts and percentage, and CD3+/CD8+ T-cells and percentage**

Months after thymectomy	Absolute lymphocyte count (mcL)	CD3+/CD4+ absolute (mm ^3^)	CD3+/CD4+ percentage	CD3+/CD8+ absolute (mm ^3^)	CD3+/CD8+ percentage
**8**	685	75	10.9	336	49.0
**9**	755	85	11.3	366	49.0
**13**	912	98	10.8	434	47.6
**16**	737	87	11.8	345	46.8
**19**	851	102	12.0	369	43.4
**24**	1318	130	9.9	554	42.0
**31**	1281	128	10.0	461	36.0
**36**	1321	136	10.4	428	32.6
**42**	1235	126	10.2	378	30.6
**54**	1060	160	15.1	306	28.9
**60**	888	131	14.0	253	26.0
**67**	774	145	18.0	227	28.0
**79**	846	160	18.0	241	27.0
**85**	956	164	16.0	257	25.0
Age-appropriate levels	850–3900	490–1740	30–61	180–1170	12–42

All of our patient’s laboratory values are arranged by month post-thymectomy. Laboratory values include absolute lymphocyte count, CD3+/CD4+ absolute count, CD3+/CD4+ percentage, CD3+/CD8+ absolute count, and CD3+/CD8+ percentage. Age-appropriate laboratory values have been provided in the last row.

One year later and a total of 3 years after his thymectomy, his yearly positron emission tomography/computed tomography (PET/CT) scan revealed a small posteriorly located right apical lung nodule with mild metabolic activity. A needle biopsy of the nodule was inconclusive. However, a PET/CT scan a year later showed that the mass had increased by 3mm. As a result, he received a right thoracoscopy and wedge resection of the nodule in his right lung, a total of 47 months since his thymectomy. Analysis of the nodule revealed that it was benign, yet it was inflammatory in nature with granulomatous features.

**Figure 1:**
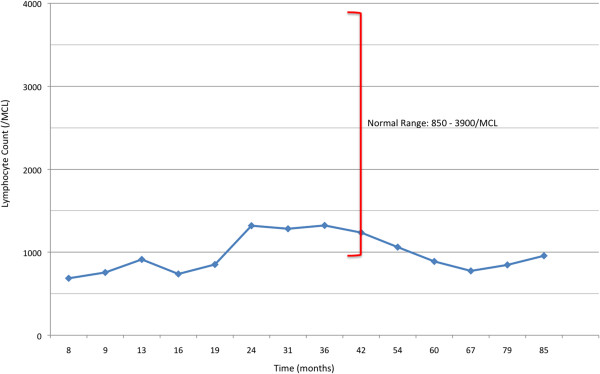
**Absolute lymphocyte count over 7-year period.** The patient’s absolute lymphocyte counts measured in cells/mcL are shown based on the amount of time in months post-thymectomy. The blue data points represent the patient’s lymphocyte counts. Age-appropriate lymphocyte counts are represented by the red vertical bracket, with normal values provided to the right of the bracket.

**Figure 2:**
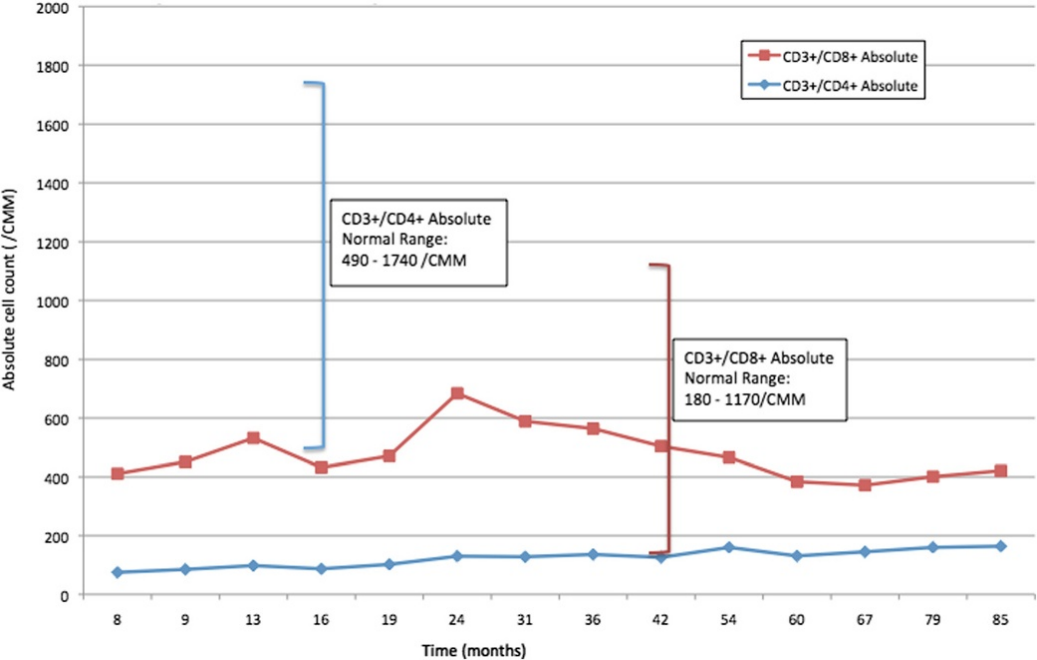
**CD3+/CD4+ and CD3+/CD8+ absolute counts over 7-year period.** The patient’s absolute T-cell counts of CD3+/CD4+ and CD3+/CD8+ measured in cells/mm^3^ are shown based on the amount of time in months post-thymectomy. The red data points represent CD3+/CD8+ absolute cell counts; the blue data points represent CD3+/CD4+ absolute cell counts. Age-appropriate T-cell counts are represented by the vertical brackets, with values provided in the adjacent boxes. The red bracket corresponds to normal CD3+/CD8+ absolute cell counts, and the blue bracket corresponds to normal CD3+/CD4+ absolute cell counts.

In the following 3 years since the lung resection, we monitored him with a physical examination every 2 to 3 months and a laboratory work-up every 4 to 7 months. He continued to complain of hives consistent with CIU. During this time, he maintained his drug plan of doxepin 50mg every evening at bedtime, fexofenadine 180mg once daily before noon, and Bactrim^®^ (trimethoprim-sulfamethoxazole) double strength once daily prophylaxis. As in the months before, he experienced CD4+ lymphocytopenia, wavering between approximately 130/mm^3^ and 160/mm^3^. During the 3 years preceding his last visit, he experienced no recurrent sinopulmonary or opportunistic infections for 3 years while maintaining Bactrim^®^ (trimethoprim-sulfamethoxazole) prophylaxis. This allowed for discontinuation of the prophylaxis treatment, contingent on close monitoring of his health and resumption of Bactrim^®^ (trimethoprim-sulfamethoxazole) if symptoms occurred. His last laboratory work-up at our office, approximately 85 months after his thymectomy, revealed an absolute lymphocyte count of 956 cells/mcL, absolute CD3+/CD4+ of 164/mm^3^ (16%), and absolute CD3+/CD8+ of 257/mm^3^ (25%): nml 850 to 3900 cells/mcL; 490 to 1740/mm^3^, 30 to 61%; 180 to 1170/mm^3^, 12 to 42%, respectively (See Figures 1 and 2).

## Discussion

The laboratory results from our patient suggest a stabilization of CD4+ cell counts, which is consistent with other patients who experienced similar therapies. According to research involving patients with head and neck tumors, similar cellular immune system disturbances in individual patients were noted 11 years post-radiation therapy. Our study, as well as others, shows that radiation-induced T-cell deficiency in patients may be irreversible [13]. While radiation therapy to the thymus is detrimental to the T-cell pool, thymus resection does not necessitate immunodeficiency. It has been shown that only thymectomies where the thymus was active at the time of surgery result in a marked decrease in peripheral T-cell concentration. In patients for whom thymopoiesis was minimal, there was little effect on T-cell concentration [14].

In addition to chronic T-cell lymphocytopenia, our patient also developed chronic idiopathic urticaria, a condition that we hypothesize is autoimmune. There is mounting evidence of autoimmune diseases developing in patients undergoing thymectomy with or without pre-existing MG, suggesting a role of thymectomy in the induction of autoantibodies [15–17]. However, thymectomy may exert contrasting effects on the course of distinct autoimmune diseases. In certain instances, a thymectomy may relieve the source and therefore the symptoms of a pathological condition. In other situations, thymectomy may precipitate a clinical deterioration or even create a new disease, suggesting a protective role of the thymus against autoimmunity. For example, thymoma-induced immunodeficiency, commonly known as Good syndrome, is characterized by hypogammaglobulinemia, low or absent B-cells in peripheral blood, and variable defects in cell-mediated immunity [18, 19]. The treatment for Good syndrome is to first remove the thymic tumor and to then follow up with immunoglobulin replacement [20]. However, unlike our patient, patients with Good syndrome do not manifest with severe CD4+ lymphocytopenia. Our patient’s lymphocytopenia, as well as his development of autoimmune symptoms following thymus resection, suggest that our patient’s thymectomy in conjunction with his radiation therapy, are likely culprits for immunodeficiency, and not his thymoma.

Another possible explanation for our patient’s condition is that he had a pre-existing autoimmune disorder and later developed thymoma. The most common autoimmune disease diagnosed in patients who later develop thymoma is MG, while less likely conditions in this setting are SLE, pure red blood cell aplasia, hypogammaglobulinemia, rheumatoid arthritis, and dermatomyositis [21]. While we cannot be certain our patient did not have some subclinical manifestation of any number of these conditions, it is highly unlikely given his medical records and history.

Our patient did not have any immune work-up after being diagnosed with thymoma, or immediately after his thymectomy and radiation treatment. We do not know if he had immune defects as a result of the thymoma, or if he developed the severe CD4+ lymphocytopenia entirely secondary to thymectomy and/or radiation. Although our patient’s history appeared to be negative for immunodeficiency before his thymectomy, we advise that patients with thymoma should receive an immune work-up including CD4+ and CD8+ counts prior to treatment. Awareness of immunodeficiency in a patient may help physicians quickly identify opportunistic infections, the need for prophylaxis, and his or her tolerance for vaccines. For example, our patient was placed on Bactrim^®^ (trimethoprim-sulfamethoxazole) double strength once daily prophylaxis as a precaution to decrease the likelihood of infection in his immunodeficient state.

## Conclusions

Our patient presented with a persistent low CD4+ T-cell count for 8 years following a combination of thymectomy and radiation therapy for thymoma. This case dictates the need for an awareness of autoimmune disorders in patients with similar treatment histories. We recommend that patients with thymoma with or without thymectomy/radiation therapy should be considered for an immune work-up both prior and subsequent to treatment. Continuous monitoring of their immune dysfunction may be critical, especially within the first year of treatment. Prospective studies must be conducted to assess immunodeficiency and autoimmune disorders in patients that have had both thymectomy and radiation.

## Consent

Written informed consent was obtained from the patient for publication of this case report. A copy of the written consent is available for review by the Editor-in-Chief of this journal.

## Abbreviations

CIU: Chronic idiopathic urticaria
MG: Myasthenia gravis
nml: Normal values
PET/CT: Positron emission tomography/computed tomography
SLE: Systemic lupus erythematosus
TSH: Thyroid-stimulating hormone.
